# Early acetaminophen use is associated with the reduced mortality risk in patients with sepsis-associated encephalopathy: a retrospective study

**DOI:** 10.1186/s40001-025-02786-y

**Published:** 2025-06-23

**Authors:** Fengzhen Huang, Jiping Yi, Tieqiao Zhou, Xiaoxiang Gong

**Affiliations:** 1 Department of Neurology, The First People’s Hospital of Chenzhou affiliated to the University of South China, Chenzhou, 423000 Hunan China; 2 Department of Neurology, The First Affiliated Hospital of Xiangnan University, Chenzhou, 423000 Hunan China; 3 Department of Laboratory Medicine, The First People’s Hospital of Chenzhou affiliated to the University of South China, Chenzhou, 423000 Hunan China; 4 Department of Pediatrics, The Second Xiangya Hospital of Central South University, Changsha, 410011 Hunan China

**Keywords:** Sepsis, Sepsis-associated encephalopathy, Acetaminophen, MIMIC IV database, Mortality

## Abstract

**Background:**

Sepsis-associated encephalopathy (SAE), a severe neurological complication of systemic infection, carries substantial morbidity and mortality risks. This study aims to examine the relationship between early acetaminophen use and survival rates in critically ill SAE patients.

**Methods:**

Using data from the MIMIC-IV database, we conducted a retrospective cohort study on patients with SAE, stratified by acetaminophen exposure within 48 h of ICU admission. Among the 4111 eligible patients (1689 acetaminophen recipients versus 2422 non-recipients), propensity score matching resulted in 3124 matched subjects (1562 per cohort). The primary outcome was 90-day mortality, while secondary outcomes included mortality rates at 30, 60, 180, and 365 days. Survival analyses utilized Cox proportional hazards regression and Kaplan–Meier curves, supplemented by subgroup analyses for 90-day mortality.

**Results:**

Acetaminophen exposure was correlated with reduced 30-day mortality rate (HR = 0.78, 95%CI [0.65–0.94], *p* < 0.05), as well as decreased 60-day (HR = 0.71, 95%CI [0.60–0.83], *p* < 0.001), 90-day (HR = 0.70, 95%CI [0.60–0.81], *p* < 0.001), 180-day (HR = 0.70, 95%CI [0.60–0.80], *p* < 0.001) and 365-day (HR = 0.69, 95%CI [0.61–0.79], *p* < 0.001) mortality rate after PSM. The Kaplan–Meier analysis demonstrated significantly higher survival rates in the acetaminophen group compared to the non-acetaminophen group, with a persistent trend at 30, 90, 180, and 365 days (log-rank *p* < 0.05). The protective effect was consistent across subgroups except acetaminophen dosage ≥ 650 mg.

**Conclusion:**

Early administration of acetaminophen is associated with reduced short- and long-term mortality in SAE patients. These findings support a potential therapeutic role for acetaminophen in SAE and warrant further mechanistic and prospective validation.

**Supplementary Information:**

The online version contains supplementary material available at 10.1186/s40001-025-02786-y.

## Introduction

Sepsis-associated encephalopathy (SAE) is one of the common complications in sepsis patients, which is characterized by decreased level of consciousness, cognitive dysfunction, and neuropsychiatric symptoms [1]. It is estimated that up to 70% of sepsis patients develop SAE, imposing a heavy burden on the healthcare system [2]. The development of SAE involves a variety of pathophysiological mechanisms, including systemic inflammatory response, oxidative stress, blood–brain barrier damage, neuronal dysfunction, and abnormal cerebral perfusion [3, 4]. Systemic inflammation due to sepsis can activate the neuroinflammatory response through pro-inflammatory cytokines (e.g., IL-6, TNF-α), which can further aggravate brain damage [5]. In addition, the increased permeability of the blood–brain barrier allows harmful substances to enter the central nervous system, leading to neuronal apoptosis and synaptic dysfunction [6]. The hypoperfusion state and hypoxic injury may also further deteriorate neurologic function [7, 8]. Despite the optimization of sepsis management strategies in recent years, the targeted treatment of SAE is still extremely limited, and its clinical management faces great challenges.

In recent years, more and more studies have focused on the effect of different drugs on the prognosis of sepsis [9, 10]. Acetaminophen is a drug commonly used to reduce fever in sepsis, and there has been some research exploring its effects beyond fever reduction. Ouellet M have shown that acetaminophen inhibits hemoglobin-mediated lipid peroxidation by reducing iron protoporphyrin radicals in cell-free hemoglobin (CFH) [11]. In animal experiments, acetaminophen has been shown to protect against oxidative damage and kidney failure resulting from the release of myoglobin triggered by rhabdomyolysis [12]. In an observational study involving adult patients with severe sepsis, acetaminophen was associated with decreased oxidative damage and a reduction in in-hospital mortality [13]. In sepsis-associated diseases, acetaminophen has been shown to reduce the inflammatory response and exhibit some protective effects in sepsis-associated acute kidney injury (SA-AKI) [14]. In patients with septic and septic shock, it may improve vascular function by reducing circulating levels of inflammatory factors, which in turn improves the perfusion of organs [15]. Some studies have also suggested that acetaminophen may have a potential protective effect against sepsis-related multiple organ dysfunction syndrome (MODS) [16]. While some studies have examined the role of acetaminophen in sepsis, the effect of acetaminophen on the prognosis of SAE is entirely unknown.

Recent studies on animals indicate that acetaminophen may possess certain neuroprotective effects. Zhao et al. found that acetaminophen may protect against LPS-induced cognitive dysfunction by reducing inflammation, oxidative stress, and mitochondrial-mediated apoptosis through its antioxidant and anti-inflammatory effects [17]. The study of isolated brain microvessels from rats indicated that acetaminophen could be an effective agent for preserving cerebrovascular function [18]. The addition of acetaminophen alleviated the decrease in vascular endothelial growth factor (VEGF) and pigment epithelium-derived factor (PEDF), which significantly diminish with age and are crucial for maintaining neuronal viability. Utilizing the nematode Caenorhabditis elegans as a model organism, researchers discovered that acetaminophen provides significant protection to the dopamine neurons of C. elegans against stressors associated with oxidative damage [19]. Additionally, two studies conducted on septic mice have demonstrated that acetaminophen suppresses ferroptosis in the hippocampus and enhances cognitive function [20, 21]. However, whether acetaminophen has a specific protective effect against SAE, a complication characterized by neuroinflammation and blood–brain barrier disruption, has not been clarified.

As early intervention is considered critical in SAE, we conducted a retrospective cohort study using the MIMIC-IV database to evaluate the association between early acetaminophen use and mortality at 30 days, 60 days, 90 days, 180 days, and 365 days in patients with SAE. To be more specific, our hypothesis was that the early use of acetaminophen would reduce the mortality rate of SAE compared to patients who did not receive it.

## Methods

### Data source

This study utilized clinical data from the MIMIC-IV database (version 3.1), a publicly available critical care dataset containing de-identified health information from Intensive care unit (ICU) admissions at the Beth Israel Deaconess Medical Center between 2008 and 2022 [22, 23]. The database has been approved by the Institutional Review Boards (IRBs) of both the Massachusetts Institute of Technology (MIT) and Beth Israel Deaconess Medical Center. Given the retrospective design and the use of fully anonymized data, the requirement for individual informed consent was waived, and no additional ethical approval was required. Access to the database was granted after the completion of the necessary training and credentialing process (Fengzhen Huang, Certification IDs: 63,858,817 and 63,858,818).

### Study population

Sepsis was characterized by suspected infection concurrent with a sepsis-related organ failure assessment (SOFA) score ≥ 2 [24]. Diagnosis of SAE required fulfillment of three criteria: (1) meeting diagnostic standards of sepsis; (2) exhibiting either delirium or a Glasgow Coma Scale (GCS) score < 15; and (3) Ruling out disturbances of consciousness attributed to other identifiable alternative causes encompasses: primary neurological conditions such as cerebrovascular disorders, CNS infections, epilepsy, or brain neoplasms; pre-existing neuropsychiatric comorbidities or substance use disorders; metabolic encephalopathy, hepatic encephalopathy, hypertensive encephalopathy, and other hepatic or renal disorders that impact consciousness; and severe electrolyte imbalances (serum sodium < 120 mmol/L or > 150 mmol/L) or glucose dysregulation (< 54 mg/dL or > 180 mg/dL) [25]. The ruling-out list has been added to Supplementary Table S1–S9. To establish a diagnosis of delirium, a patient must have a Richmond Agitation-Sedation Scale (RASS) score of −3 or above, while patients with a RASS score below −3 are identified as comatose and therefore excluded from further assessment [26]. Those scoring − 3 or higher underwent evaluation using the Confusion Assessment Method for the ICU (CAM-ICU). Patients were considered to have a delirious status with evidence of acute or fluctuating changes in mental status (Criterion 1), inattention (Criterion 2), and either disorganized thinking (Criterion 3) or an altered level of consciousness (Criterion 4) [27, 28]. In our study, SAE was defined as sepsis accompanied by a GCS score of less than 15 or delirium within 24 h of ICU admission. For patients undergoing analgesia, sedation, or endotracheal intubation, the GCS scores were assessed prior to these interventions. For each individual, we used the lowest GCS score recorded within the first 24 h of ICU admission. For a limited number of patients whose GCS scores were truly unavailable, we excluded them. Eligible SAE participants included adults (≥ 18 years) with initial ICU admissions lasting ≥ 24 h. Exclusion criteria included datasets with more than 15% missing variables.

### Clinical variables

Collected demographic variables included age, sex, race, and body weight. Laboratory metrics encompassed white blood cell count (WBC), platelets, glucose, serum sodium, creatinine, and blood urea nitrogen (BUN). Disease severity evaluations incorporated SOFA score, GCS score, and Charlson Comorbidity Index (CCI) score. Extracted vital signs comprised average peripheral oxygen saturation (SpO2), heart rate, respiratory (Resp) rate, body temperature, systolic blood pressure (SBP), and diastolic blood pressure (DBP). Laboratory data and were collected within the first 24 h of hospitalization, and vital signs were averaged over the first 24 h of ICU admission. The documented therapeutic interventions included vasoactive agents, aspirin, dexmedetomidine, and haloperidol. The comorbidity profile covered hypertension, diabetes mellitus, cardiovascular disorders, and chronic obstructive pulmonary disease (COPD). Data retrieval was conducted via Structured Query Language (SQL).

### Outcomes

Patients were divided into two groups: one for acetaminophen and one for nonacetaminophen. Acetaminophen administration within the first 48 h of ICU admission was considered acetaminophen exposure. Acetaminophen intake routes include oral, nasal, and rectal administration. The primary outcome was mortality as measured at 90 days. 30-day mortality, 60-day mortality, 180-day mortality, and 365-day mortality served as secondary outcomes. We also compared the length of time in hospital, length of time in ICU and duration of ventilation. To identify the possible effect of acetaminophen in patients with SAE, we investigated its effect on these outcomes. Stratified subgroup analyses were conducted to examine outcome variability across demographic and clinical subgroups.

### Statistical analysis

Statistical analyses were performed using R software (version 4.4.0), with missing data randomized through the “mice” package. Continuous variables that follow a normal distribution are expressed as mean ± standard deviation and are compared across groups using *t*-tests. For continuous variables with a skewed distribution, data are presented as median and interquartile range, and group differences were assessed with the Mann–Whitney *U* test. Categorical variables were evaluated using the chi-square test. Propensity score matching (PSM) with a 1:1 nearest neighbor algorithm and a caliper width of 0.05 was applied to balance baseline characteristics between the cohorts [29]. The balance of the clinical variables was quantified using standardized mean differences (SMD), with an SMD less than 0.1 indicating adequate balance. The proportional hazards (PH) assumption was formally evaluated using the Schoenfeld residuals test. When the assumption was not violated (*p* ≥ 0.05), Cox proportional hazards regression models were employed to estimate the association between acetaminophen exposure and mortality risk, with results presented as hazard ratios (HRs) and corresponding 95% confidence intervals (CIs). In instances where the PH assumption was violated (*p* < 0.05), piecewise Cox regression models were utilized to account for time-dependent effects. Group-specific survival probabilities were visualized through Kaplan–Meier curves and statistically compared using log-rank test. E-value analysis was conducted to evaluate the possible influence of unmeasured or unknown confounders, aiming to estimate the magnitude of confounding required to nullify the observed link between acetaminophen administration and mortality risk [30]. Statistical significance was defined as a two-sided p-value less than 0.05.

## Results

### Baseline characteristics

**Fig. 1:**
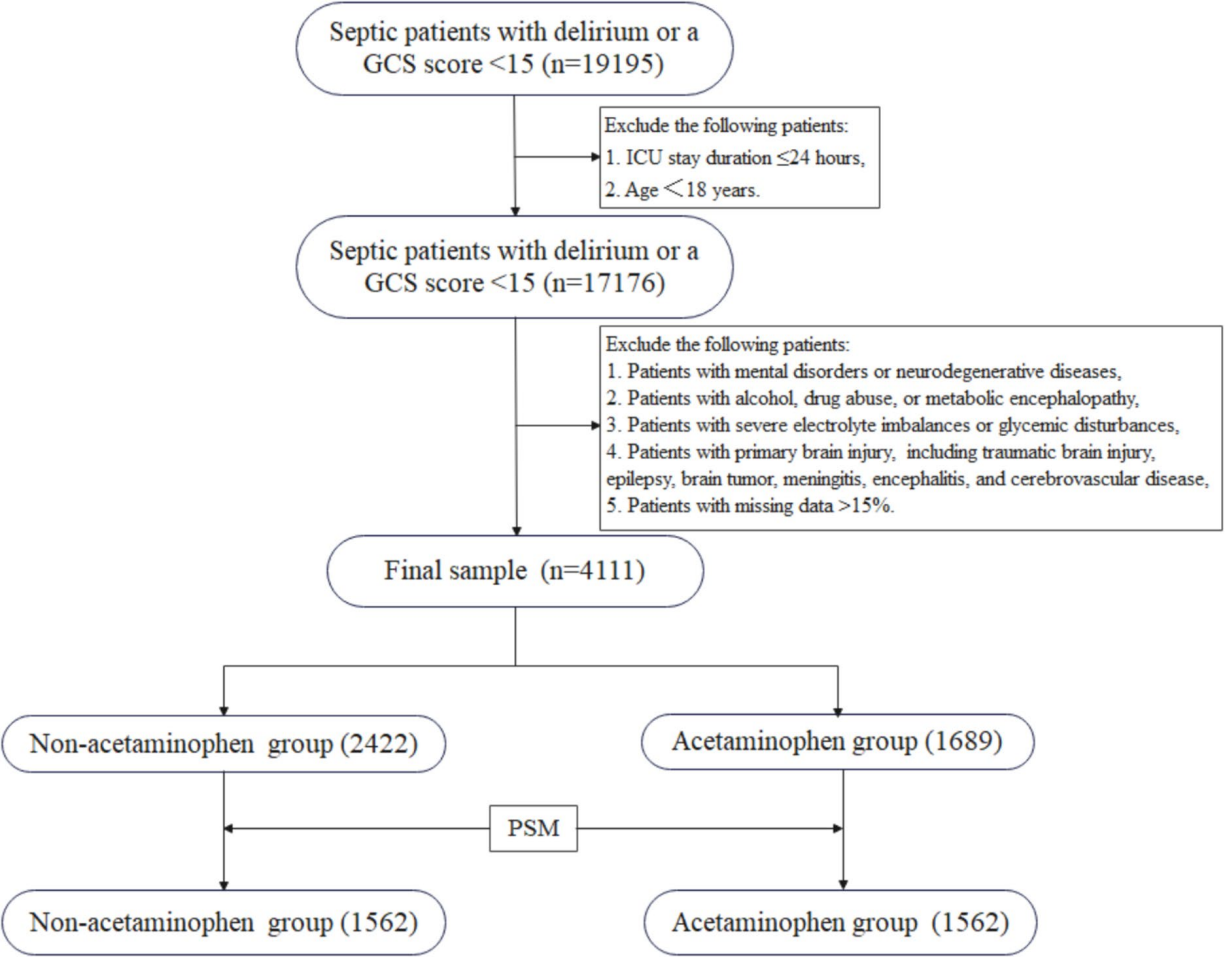
The flowchart for the selection of SAE patients. GCS: Glasgow Coma Scale, SAE: sepsis-associated encephalopathy, ICU: intensive care unit

**Fig. 2:**
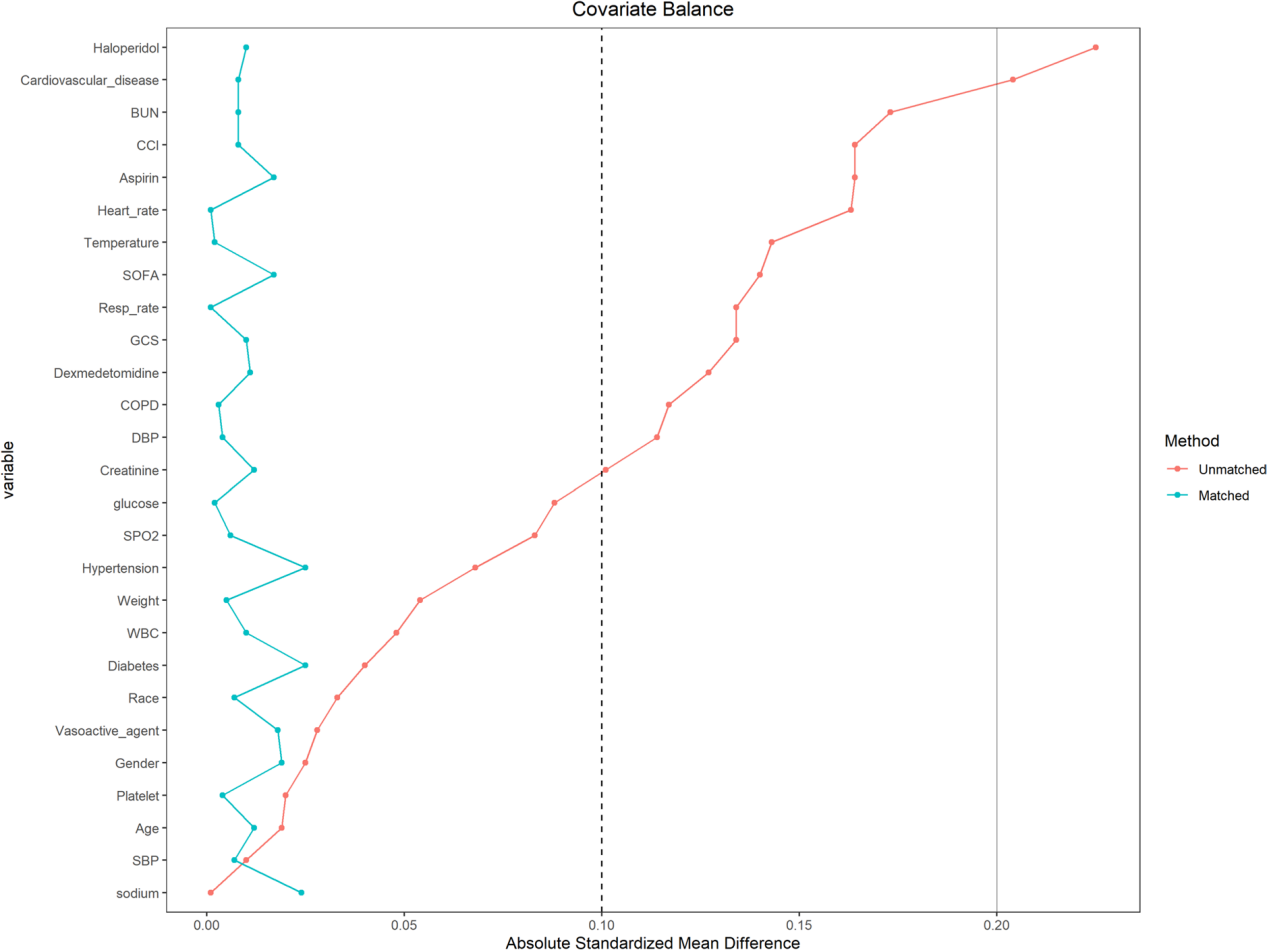
Standardized mean difference (SMD) of variables before and after propensity score matching (PSM)

The patient selection process is presented in Fig. 1. We selected septic patients with delirium or a GCS score less than 15 at the first 24 h after admission in ICU, with 19,195 diagnosed with SAE. After excluding patients younger than 18 years or those with an ICU stay duration of less than 24 h, 17,176 patients were included. Finally, 4111 patients were ultimately selected after the exclusion criteria. Among these individuals, 2422 did not receive acetaminophen therapy and were classified as the non-acetaminophen group, while 1689 patients received acetaminophen treatment and were categorized as the acetaminophen group. As illustrated in Table 1, compared to the non-acetaminophen group, patients in the acetaminophen group exhibited higher body weight, slightly lower WBC levels, glucose levels, creatinine levels, and BUN levels. Patients in the acetaminophen group also had higher body temperatures and lower heart rates, respiratory rates, and DBP. A higher proportion of these patients received aspirin, while a lower proportion received dexmedetomidine and haloperidol treatment. Additionally, a lower proportion of patients in the acetaminophen group had diabetes. After PSM, both the non-acetaminophen group and the acetaminophen group contained of 1562 patients each. The groups were well matched, with SMD all < 0.1 and p-values > 0.05. Figure 2 shows the comparison of baseline characteristics between the groups before and after PSM.

**Table 1:** Baseline characteristics of SAE patients before and after PSM

Variables	Before PSM					After PSM				
	Overall (4111)	Non-acetaminophen (2422)	Acetaminophen (1689)	*p*	SMD	Overall (3124)	Non-acetaminophen (1562)	Acetaminophen (1562)	*p*	SMD
Demographics										
Age (median [IQR])	71.2 [59.9, 81.0]	71.3 [60.0, 81.0]	70.9 [59.9, 81.1]	0.696	0.019	71.2 [59.7, 81.1]	71.3 [59.8, 81.0]	71.1 [59.7, 81.3]	0.969	0.012
Gender (%)										
Female	1729 (42.06)	1031 (42.57)	698 (41.33)	0.446	0.025	1327 (42.48)	656 (42.00)	671 (42.96)	0.612	0.019
Male	2382 (57.94)	1391 (57.43)	991 (58.67)			1797 (57.52)	906 (58.00)	891 (57.04)		
Race (%)										
White	2914 (70.88)	1702 (70.27)	1212 (71.76)	0.319	0.033	2239 (71.67)	1117 (71.51)	1122 (71.83)	0.874	0.007
Other	1197 (29.12)	720 (29.73)	477 (28.24)			885 (28.33)	445 (28.49)	440 (28.17)		
Weight (median [IQR])	77.0 [65.0, 92.0]	76.2 [64.0, 91.7]	77.8 [65.9, 92.2]	0.015	0.054	77.0 [64.9, 92.0]	77.0 [64.0, 92.0]	77.0 [65.0, 91.1]	0.503	0.005
Laboratory index										
WBC (median [IQR])	9.8 [7.0, 14.5]	10.0 [7.0, 14.8]	9.6 [6.9, 13.9]	0.023	0.048	9.6 [7.0, 14.1]	9.6 [6.9, 14.1]	9.6 [7.0, 14.1]	0.979	0.01
Platelet (median [IQR])	203.0 [146.0, 274.0]	204.0 [145.0, 276.0]	201.0 [148.0, 271.0]	0.962	0.02	204.0 [148.0, 274.0]	204.0 [148.0, 274.0]	202.0 [148.0, 274.8]	0.958	0.004
Glucose (median [IQR])	116.0 [98.0, 138.0]	117.0 [99.0, 139.0]	115.0 [97.0, 136.0]	0.005	0.088	115.0 [97.0, 137.0]	115.0 [98.0, 136.8]	115.0 [97.0, 137.0]	0.944	0.002
Sodium (median [IQR])	139.0 [136.0, 141.0]	139.0 [136.0, 141.0]	139.0 [136.0, 141.0]	0.842	0.001	139.0 [136.0, 141.0]	139.0 [136.0, 141.0]	139.0 [136.0, 141.0]	0.565	0.024
Creatinine (median [IQR])	1.0 [0.8, 1.6]	1.0 [0.8, 1.7]	1.0 [0.8, 1.4]	0.015	0.101	1.0 [0.7, 1.5]	1.0 [0.7, 1.5]	1.0 [0.8, 1.5]	0.653	0.012
BUN (median [IQR])	21.0 [14.0, 36.0]	22.0 [15.0, 38.0]	20.0 [14.0, 31.0]	< 0.001	0.173	21.0 [14.0, 34.0]	21.0 [14.0, 34.0]	21.0 [14.0, 33.0]	0.542	0.008
Severity score										
SOFA (median [IQR])	3.0 [2.0, 4.0]	3.0 [2.0, 4.0]	3.0 [2.0, 4.0]	< 0.001	0.14	3.0 [2.0, 4.0]	3.0 [2.0, 4.0]	3.0 [2.0, 4.0]	0.563	0.017
GCS (median [IQR])	14.0 [10.0, 14.0]	14.0 [10.0, 14.0]	14.0 [11.0, 14.0]	< 0.001	0.134	14.0 [11.0, 14.0]	14.0 [11.0, 14.0]	14.0 [11.0, 14.0]	0.264	0.01
CCI (median [IQR])	5.0 [3.0, 7.0]	5.0 [3.0, 7.0]	5.0 [3.0, 7.0]	< 0.001	0.164	5.0 [3.0, 7.0]	5.0 [3.0, 7.0]	5.0 [3.0, 7.0]	0.614	0.008
Vital signs										
Temperature (median [IQR])	36.8 [36.6, 37.1]	36.8 [36.5, 37.1]	36.8 [36.6, 37.1]	0.002	0.143	36.8 [36.6, 37.1]	36.8 [36.6, 37.1]	36.8 [36.6, 37.1]	0.575	0.002
Heart_rate (median [IQR])	86.1 [76.0, 97.4]	87.1 [76.1, 99.0]	84.6 [75.9, 94.7]	< 0.001	0.163	84.8 [75.1, 95.8]	84.6 [74.4, 96.4]	84.8 [75.9, 95.3]	0.709	0.001
Resp_rate (median [IQR])	19.1 [16.7, 22.1]	19.4 [16.8, 22.5]	18.8 [16.5, 21.7]	< 0.001	0.134	19.0 [16.5, 21.8]	18.9 [16.6, 21.8]	19.0 [16.5, 21.9]	0.866	0.001
SBP (median [IQR])	112.0 [104.0, 122.5]	111.9 [103.6, 123.2]	112.1 [104.5, 121.9]	0.439	0.01	112.1 [104.1, 122.5]	112.0 [104.1, 123.0]	112.3 [104.2, 122.3]	0.557	0.007
DBP (median [IQR])	59.6 [53.8, 66.2]	60.1 [53.6, 66.9]	59.0 [53.9, 64.7]	0.005	0.114	59.4 [53.6, 65.7]	59.4 [53.1, 66.0]	59.3 [53.9, 65.2]	0.841	0.004
SPO2 (median [IQR])	97.0 [95.6, 98.3]	97.0 [95.5, 98.3]	97.1 [95.8, 98.2]	0.143	0.083	97.1 [95.7, 98.3]	97.1 [95.7, 98.4]	97.0 [95.8, 98.2]	0.613	0.006
Therapy										
Vasoactive_agent (%)	1896 (46.12)	1131 (46.70)	765 (45.29)	0.392	0.028	1402 (44.88)	708 (45.33)	694 (44.43)	0.64	0.018
Aspirin (%)	2061 (50.13)	1133 (46.78)	928 (54.94)	< 0.001	0.164	1641 (52.53)	814 (52.11)	827 (52.94)	0.667	0.017
Dexmedetomidine (%)	622 (15.13)	411 (16.97)	211 (12.49)	< 0.001	0.127	414 (13.25)	210 (13.44)	204 (13.06)	0.792	0.011
Haloperidol (%)	627 (15.25)	448 (18.50)	179 (10.60)	< 0.001	0.225	363 (11.62)	184 (11.78)	179 (11.46)	0.823	0.01
Comorbidities										
Hypertension (%)	1606 (39.07)	913 (37.70)	693 (41.03)	0.034	0.068	1233 (39.47)	607 (38.86)	626 (40.08)	0.51	0.025
Diabetes (%)	1082 (26.32)	655 (27.04)	427 (25.28)	0.22	0.04	793 (25.38)	388 (24.84)	405 (25.93)	0.511	0.025
Cardiovascular_disease (%)	1203 (29.26)	616 (25.43)	587 (34.75)	< 0.001	0.204	990 (31.69)	492 (31.50)	498 (31.88)	0.848	0.008
COPD (%)	232 (5.64)	163 (6.73)	69 (4.09)	< 0.001	0.117	139 (4.45)	70 (4.48)	69 (4.42)	1	0.003

WBC: white blood cell, BUN: blood urea nitrogen, SOFA: sepsis-related organ failure assessment, GCS: glasgow coma scale, CCI: charlson comorbidity index, Resp: respiration, SBP: systolic blood pressure, DBP: diastolic blood pressure, SPO2: saturation of peripheral oxygen, COPD: chronic obstructive pulmonary disease

### Acetaminophen exposure and clinical outcomes

Table 2 shows the comparison of clinical outcomes between the acetaminophen and non-acetaminophen groups. The Mann–Whitney *U* test results indicated that the median length of hospital stay was significantly shorter in the acetaminophen group compared to the non-acetaminophen group, both before PSM (7.06 vs 9.82, *p* < 0.001) and after PSM (7.10 vs 9.47, *p* < 0.001). This trend was also observed in the length of time spent in the ICU, with shorter durations in the acetaminophen group before PSM (2.30 vs 3.25, *p* < 0.001) and after PSM (2.33 vs 3.00, *p* < 0.001). Furthermore, we assessed the duration of ventilation in both groups. The results indicated that patients in the acetaminophen group had a shorter ventilation time compared to the non-acetaminophen group, both before PSM (18.23 vs 22.71, *p* < 0.001) and after PSM (19.00 vs 21.00, *p* < 0.05).

**Table 2:** Composite outcomes of the acetaminophen group and non-acetaminophen group

Before PSM				
Outcome	Overall (4111)	Non-acetaminophen group (2422)	Acetaminophen group (1689)	*p*
los_hospital (median [IQR])	8.50 [5.38, 4.24]	9.82 [6.01, 16.92]	7.06 [4.93, 10.72]	< 0.001
los_icu (median [IQR])	2.87 [1.76, 5.12]	3.25 [1.96, 6.17]	2.30 [1.48, 4.01]	< 0.001
Duration_ventilation (median [IQR])	20.50 [9.57, 2.09]	22.71 [10.28, 47.48]	18.23 [8.93, 36.00]	< 0.001

After PSM				
Outcome	Overall (3124)	Non-acetaminophen group (1562)	Acetaminophen group (1562)	*p*
los_hospital (median [IQR])	8.06 [5.22, 3.17]	9.47 [5.87, 15.93]	7.10 [4.93, 10.78]	< 0.001
los_icu (median [IQR])	2.66 [1.69, 4.67]	3.00 [1.86, 5.28]	2.33 [1.51, 4.07]	< 0.001
Duration_ventilation (median [IQR])	20.00 [9.30, 9.75]	21.00 [10.00, 42.33]	19.00 [9.00, 36.65]	0.002

### Relationship between acetaminophen use and mortality

Table 3 presented the mortality between the non-acetaminophen group and the acetaminophen group. As the Schoenfeld residuals test showed no violation of the assumption (*p* > 0.05), the cox proportional hazards model was applied. The univariate analyses of cox proportional hazards model after PSM showed that acetaminophen could significantly reduce the mortality rate. The multivariate cox hazard analyses also demonstrated that patients in the acetaminophen group experienced significantly decreased 30-day mortality rate (HR = 0.78, 95%CI [0.65–0.94], *p* < 0.05), as well as lower 60-day (HR = 0.71, 95%CI [0.60–0.83], *p* < 0.001), 90-day (HR = 0.70, 95%CI [0.60–0.81], *p* < 0.001), 180-day (HR = 0.70, 95%CI [0.60–0.80], *p* < 0.001) and 365-day (HR = 0.69, 95%CI [0.61–0.79], *p* < 0.001) mortality rate. Interestingly, the impact of acetaminophen was also significant in three models before PSM, suggesting that the benefit of acetaminophen in reducing mortality was robust.

**Table 3:** Association between acetaminophen therapy and mortality of SAE patients

Outcome	Before PSM						After PSM					
	Total (4111)	Non-acetaminophen (2422)	Acetaminophen (1689)	model	HR (95% CI)	*p*	Total (3124)	Non-acetaminophen (1562)	Acetaminophen (1562)	model	HR (95% CI)	*p*
30-day mortality, n (%)	725 (17.6)	514 (21.2)	211 (12.5)	1	0.57 (0.48–0.66)	< 0.001	464 (14.9)	259 (16.6)	205 (13.1)	1	0.78 (0.65–0.94)	0.010
				2	0.57 (0.49–0.67)	< 0.001				2	0.78 (0.65–0.94)	0.009
				3	0.80 (0.67–0.94)	0.007				3	0.78 (0.65–0.94)	0.010
60-day mortality, n (%)	920 (22.4)	654 (27.0)	266 (15.7)	1	0.55 (0.48–0.63)	< 0.001	610 (19.5)	352 (22.5)	258 (16.5)	1	0.72 (0.61–0.84)	< 0.001
				2	0.56 (0.48–0.64)	< 0.001				2	0.71 (0.61–0.84)	< 0.001
				3	0.74 (0.64–0.85)	< 0.001				3	0.71 (0.60–0.83)	< 0.001
90-day mortality, n (%)	1027 (25.0)	732 (30.2)	295 (17.5)	1	0.54 (0.47–0.62)	< 0.001	680 (21.8)	393 (25.2)	287 (18.4)	1	0.71 (0.61–0.83)	< 0.001
				2	0.54 (0.47–0.62)	< 0.001				2	0.71 (0.61–0.82)	< 0.001
				3	0.71 (0.62–0.82)	< 0.001				3	0.70 (0.60–0.81)	< 0.001
180-day mortality, n (%)	1207 (29.4)	848 (35.0)	359 (21.3)	1	0.56 (0.49–0.63)	< 0.001	818 (26.2)	469 (30.0)	349 (22.3)	1	0.72 (0.62–0.82)	< 0.001
				2	0.56 (0.49–0.63)	< 0.001				2	0.71 (0.62–0.81)	< 0.001
				3	0.71 (0.63–0.81)	< 0.001				3	0.70 (0.60–0.80)	< 0.001
365-day mortality, n (%)	1416 (34.4)	977 (40.3)	439 (26.0)	1	0.58 (0.52–0.65)	< 0.001	984 (31.5)	560 (35.9)	424 (27.1)	1	0.72 (0.63–0.82)	< 0.001
				2	0.58 (0.52–0.65)	< 0.001				2	0.71 (0.63–0.80)	< 0.001
				3	0.72 (0.64–0.81)	< 0.001				3	0.69 (0.61–0.79)	< 0.001

Model 1: Unadjusted

Model 2: Adjust for Age, Gender, Race, Weight

Model 3: Adjust for Age, Gender, Race, Weight, Vasoactive_agent, Aspirin, Dexmedetomidine, Haloperidol, Hypertension, Diabetes, Cardiovascular_disease, COPD, WBC, Platelet, glucose, sodium, Creatinine, BUN, SOFA, GCS, Temperature, Heart_rate, Resp_rate, SBP, DBP, SPO2

**Fig. 3:**
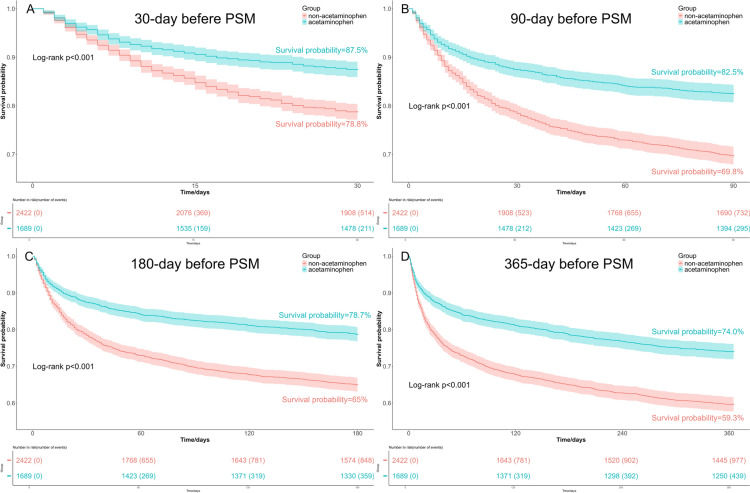
Kaplan–Meier survival curves between the acetaminophen group and non-acetaminophen group before PSM. **A** 30-day survival curves before PSM; **B** 90-day survival curves before PSM; **C** 180-day survival curves before PSM; **D** 365-day survival curves before PSM

**Fig. 4:**
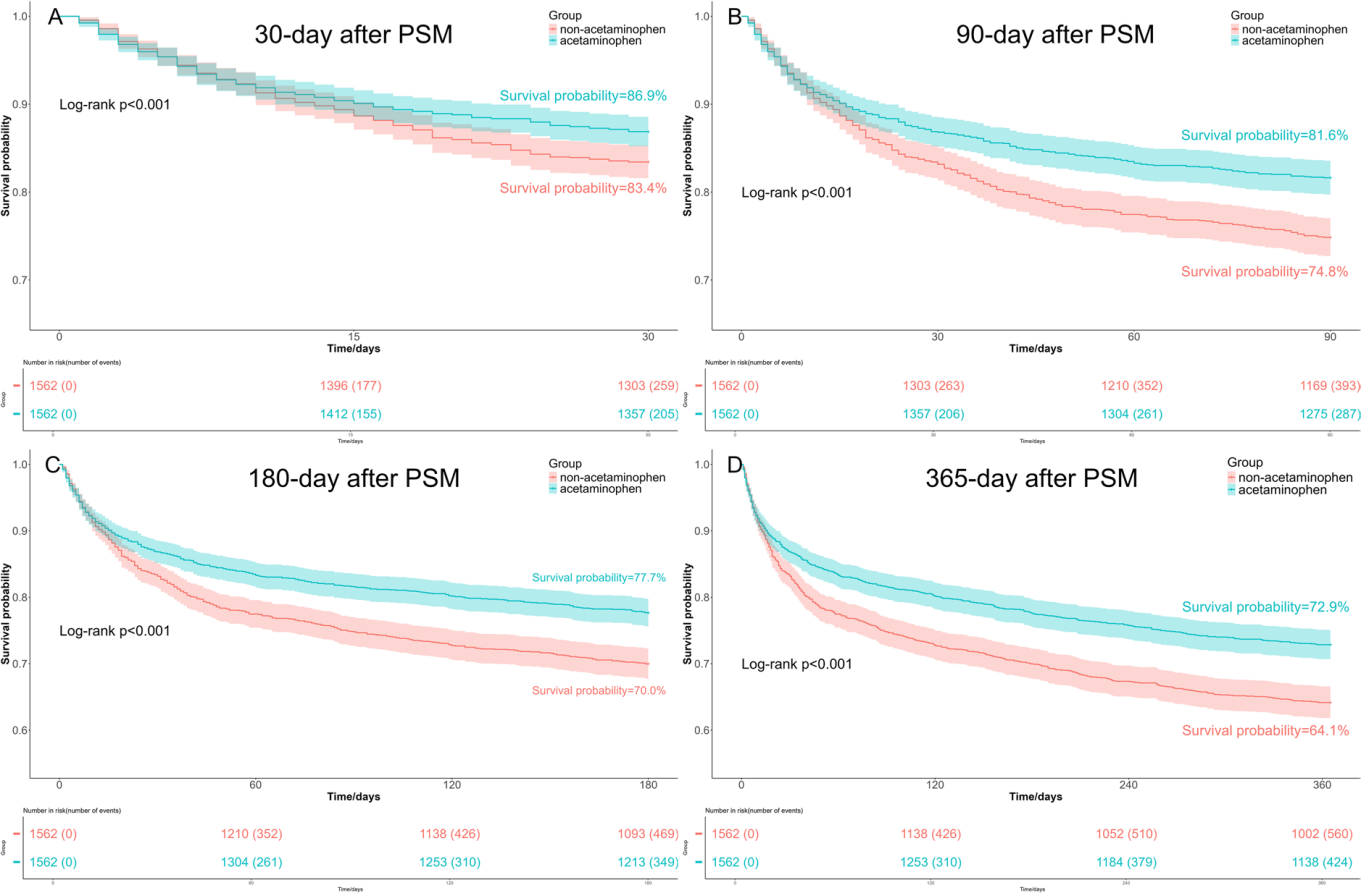
Kaplan–Meier survival curves between the acetaminophen group and non-acetaminophen group after PSM. **A** 30-day survival curves after PSM; **B** 90-day survival curves after PSM; **C** 180-day survival curves after PSM; **D** 365-day survival curves after PSM

The Kaplan–Meier curves revealed that patients received acetaminophen therapy exhibited better survival probabilities both before PSM and after PSM, compared to those who did not treated with acetaminophen. This trend persisted at 30-day, 90-day, 180-day, and 365-day, with all differences being statistically significant as indicated by a log-rank test *p*-value < 0.05 (Figs. 3 and 4).

### Subgroup analysis

**Fig. 5:**
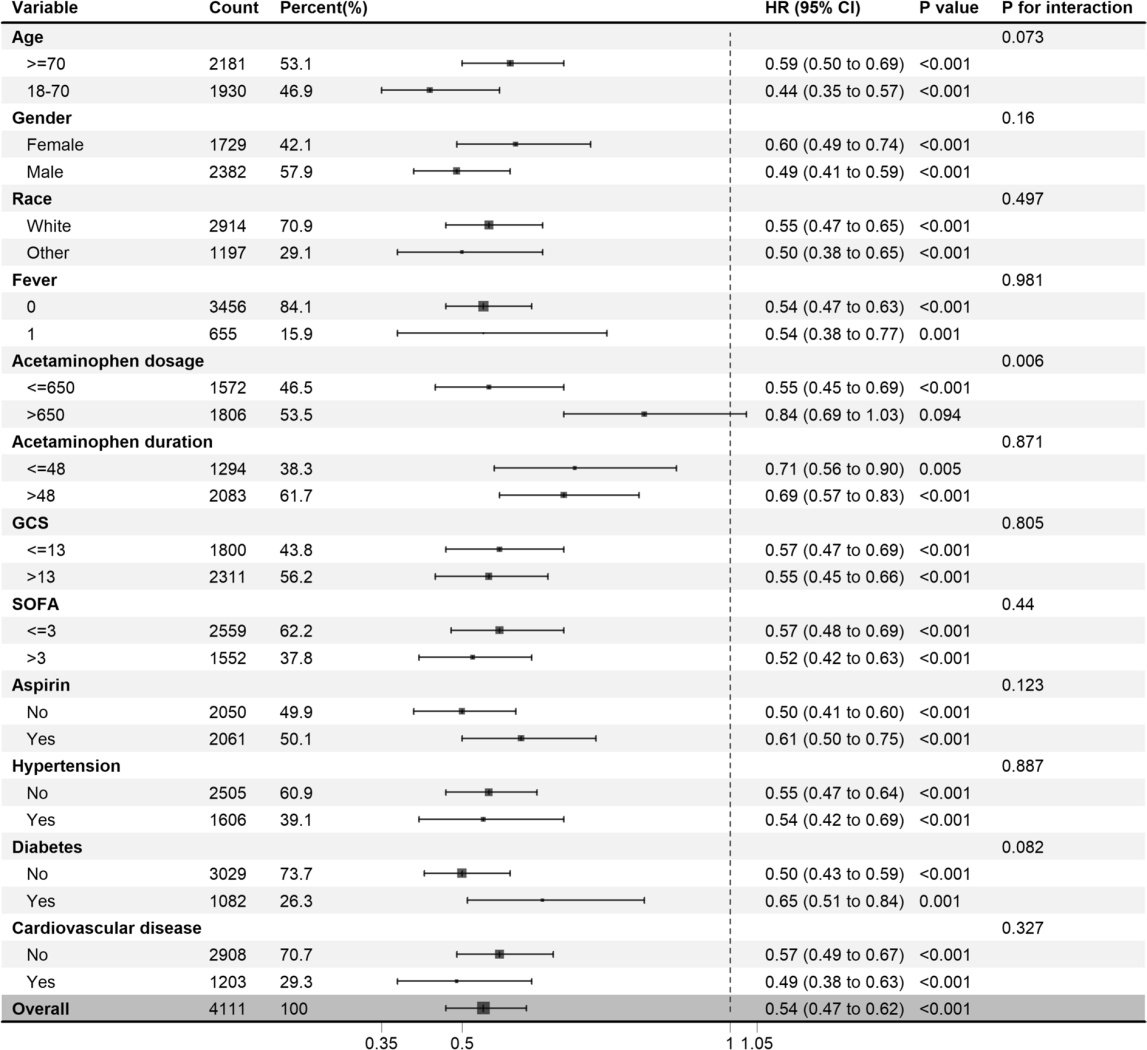
Subgroup analysis of the 90-day mortality in SAE patients. HR: hazard ratio, CI: confidence interval, SOFA: Sepsis-related Organ Failure Assessment, GCS: Glasgow Coma Scale

Acetaminophen treatment was found to significantly decrease 90-day mortality rate (HR = 0.54, 95%CI [0.47–0.62], *p* < 0.001). The protective effect of acetaminophen against SAE was consistent in subgroups divided based on age, gender, race, fever, the duration of acetaminophen, GCS score, SOFA score, aspirin adminstration, hypertension, diabetes, and cardiovascular disease. However, an interaction effect was observed in the dosage of acetaminophen, indicating different effects in subgroups. Acetaminophen was found to be protective in patients who received a total dosage of 650 mg or less (HR = 0.55, 95%CI [0.45–0.69], p < 0.05), whereas it did not demonstrate a protective effect in those administered more than 650 mg (HR = 0.84, 95%CI [0.69–1.03], *p* = 0.094) (Fig. 5).

### Sensitivity analysis

To further evaluate the potential influence of unmeasured confounding, we estimated the E-value for the association between acetaminophen therapy and mortality risk. The analysis revealed that an unmeasured confounder would require a relative risk greater than 1.88 to fully account for the observed association with 30-day mortality. This finding suggests that residual confounding is unlikely to entirely explain the association, and that any unknown or unmeasured variables are likely to have a weaker effect on 30-day mortality compared to the identified risk factors. Similar results were observed for 60-day (E-value > 2.17), 90-day (E-value > 2.21), 180-day (E-value > 2.21), and 365-day mortality (E-value > 2.26), as presented in Table 4.

**Table 4:** E-value for mortality association in acetaminophen-treated SAE patients

Outcomes	E-value	Upper limit of 95% CI
30-day mortality	1.88	1.32
60-day mortality	2.17	1.70
90-day mortality	2.21	1.77
180-day mortality	2.21	1.81
365-day mortality	2.26	1.85

## Discussion

This retrospective analysis based on the MIMIC database revealed that acetaminophen use was associated with reduced short- and long-term mortality in patients with SAE. Notably, subgroup analyses suggested that the association was attenuated at high acetaminophen dosages. This is the first study to investigate the prognostic effect of acetaminophen on patients with SAE, which is of great significance and provides a new direction for the clinical treatment of SAE.

Although acetaminophen is widely used for its antipyretic and analgesic properties, recent studies have indicated potential prognostic benefits for critically ill patients [31–33]. A multicenter retrospective research has shown that acetaminophen administration correlates with reduced in-hospital mortality among critically ill patients [34]. By analyzing data from patients with acute respiratory distress syndrome (ARDS), Wang et al. found that administering acetaminophen was associated with reduced in-hospital mortality of patients with ARDS [35]. A retrospective study conducted at the University of Virginia demonstrated that exposure to acetaminophen on the day of cardiac surgery was significantly associated with a reduced incidence of acute kidney injury [36]. An independent observational study on critically ill septic patients also indicated that acetaminophen potentially improved survival by reducing oxidative damage caused by cell-free hemoglobin [13]. Our findings are consistent with those described above, suggesting that acetaminophen use reduces short- and long-term mortality and shortens the duration of mechanical ventilation and hospitalization in SAE patients.

The mechanisms underlying the association between acetaminophen use and reduced mortality in SAE patients remain to be clarified. Current evidence suggests that acetaminophen may exert neuroprotective effects through multiple pathways: First, early administration of acetaminophen has been shown to alleviate oxidative damage in severe sepsis patients with its antioxidant properties and potentially contribute to reduced SAE mortality [37]. Animal studies further demonstrate that acetaminophen improves lipopolysaccharide (LPS)-mediated cognitive impairment by suppressing LPS-induced microglial activation and neuroinflammation in the hippocampal region [17]. Additionally, acetaminophen has been found to prevent ferroptosis in hippocampal tissues of septic mice through activation of the GPX4/FSP1 pathway, thereby reducing hippocampal damage and alleviating cognitive deficits while improving survival rates in septic models [21]. Although sepsis-induced cerebral microcirculation impairment and subsequent metabolic deterioration are recognized as significant contributors to cerebral dysfunction [7], whether acetaminophen's potential neuroprotective effects involve microcirculatory improvement in SAE requires further investigation.

Notably, a pediatric study involving 46 sepsis patients aged 7–18 years reported no association between acetaminophen and increased organ dysfunction or mortality [38]. In contrast to this pediatric cohort, our study focused exclusively on adult populations and specifically examined sepsis-induced neurological damage rather than multisystem organ failure. Furthermore, our larger sample size enhances the clinical relevance of our conclusions. An observational investigation conducted in a sepsis cohort study of 606 patients identified elevated mortality among febrile patients receiving acetaminophen [39]. A clinical trial involving 447 adults with sepsis and respiratory or circulatory organ dysfunction indicated that intravenous acetaminophen did not significantly increase the number of days alive and free of organ support in critically ill sepsis patients [16]. However, our work diverges methodologically by targeting SAE patients, allowing for a more precise evaluation of acetaminophen in distinct sepsis phenotypes. This focused approach clarifies context-specific therapeutic effects, underscoring the need for tailored management strategies in heterogeneous sepsis populations.

It is worth noting that subgroup analysis indicates a potential interaction between acetaminophen and the dosage of acetaminophen. It could be speculated that SAE patients with acetaminophen dose more than 650 mg may not benefit more from acetaminophen application. This may be related to liver injury, one of the side effects of acetaminophen, especially in critically ill patients [40]. The liver plays a key role in modulating the immune response and inflammation, and liver injury impairs these functions, making septic patients more susceptible to damage from infection and inflammation [41, 42]. In addition, liver injury can affect blood coagulation, increasing the risk of bleeding in patients with sepsis [43]. Furthermore, acetaminophen may also cause damage to the lungs and kidneys, further aggravating the severity of patients with SAE [44]. Therefore, the use of acetaminophen in patients with SAE should be carefully evaluated in terms of potential benefits and risks, and dosage should be strictly controlled. Future prospective studies or pharmacokinetic modeling approaches are warranted to validate the optimal dosing strategy for acetaminophen in SAE, and to further elucidate the balance between therapeutic benefit and potential toxicity in this population.

Although this study is based on data from a large database and has certain clinical implications, it still has some limitations. Firstly, as a retrospective study, we could not fully control the potential confounders, such as missing data inputation and other variables that may affect clinical outcomes. Secondly, the MIMIC database is primarily derived from single-center ICU data, and its sample size is limited due to PSM, which may restrict the general applicability of the results. Thirdly, SAE is a condition frequently linked with long-term neurological deficits. However, due to the lack of longitudinal neurofunctional recovery data in the MIMIC database, we were unable to assess the neurofunctional outcomes of acetaminophen on SAE. Fourthly, acetaminophen is often administered in the ICU for fever or pain, rather than specifically for SAE. Since the MIMIC database does not document the exact reasons for medication use, there may be indication-related confounding even after adjusting for illness severity and temperature. Lastly, due to the limitations of the MIMIC database, we were unable to further explore the specific mechanisms of acetaminophen. Further basic studies and prospective clinical trials are still needed to validate our findings in the future.

## Conclusions

This retrospective cohort study, based on the MIMIC database, is the first to investigate the impact of acetaminophen usage on the outcomes of SAE patients. Our findings suggest that early acetaminophen use is associated with reduced short- and long-term mortality in SAE patients. However, excessive dosing may diminish this benefit. These findings support a potential therapeutic role for acetaminophen in SAE and warrant further mechanistic and prospective validation.

## Supplementary Information

Additional file 1.

Additional file 2.

Additional file 3.

Additional file 4.

Additional file 5.

Additional file 6.

Additional file 7.

Additional file 8.

Additional file 9.

Additional file 10.

## Data Availability

The datasets presented in the current study are available in the MIMIC-IV database (https://physionet.org/content/mimiciv/3.1/). All the data generated or analyzed during this study are available upon the corresponding author on reasonable request.
