# Supplementary figures and images for: Early acetaminophen use is associated with the reduced mortality risk in patients with sepsis-associated encephalopathy: a retrospective study
Source: Eur J Med Res. 2025 Jun 23;30:512. doi: 10.1186/s40001-025-02786-y (PMC12183892; doi:10.1186/s40001-025-02786-y)

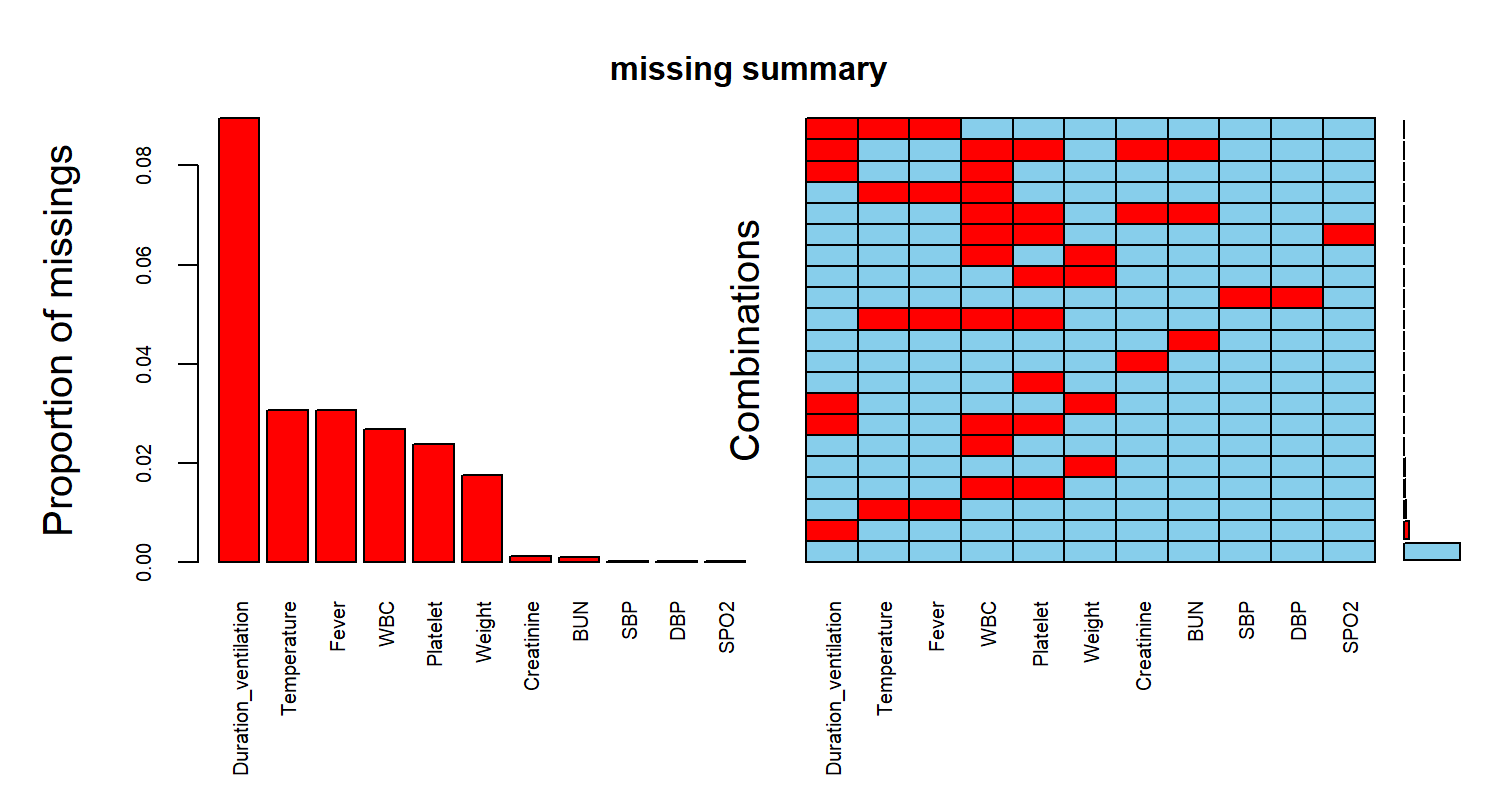

Supplement: Supplementary file 2 — Additional file 2. [file 40001_2025_2786_MOESM2_ESM.tiff]
